# The Effect of Dietary Supplements on the Quality of Life of Retired Professional Football Players

**DOI:** 10.5539/gjhs.v5n2p13

**Published:** 2012-11-22

**Authors:** Robert A. Sinnott, Rolando L. Maddela, Sejong Bae, Talitha Best

**Affiliations:** 1 Research and Development, Mannatech Incorporated, Coppell, USA; 2 Department of Biostatistics, UNTHSC, School of Public Health, Fort Worth, USA; 3 Nutritional Physiology Research Centre, Sansom Institute for Health Research, University of South Australia, Adelaide, South Australia

**Keywords:** QOL, dietary supplement, football player, professional athlete

## Abstract

Professional football players may experience negative health consequences when they retire such as chronic pain, cognitive problems as well as other consequences of sports-related injuries. The purpose of this pilot study is to determine the effects of dietary supplementation with multiple nutrients on the quality of life of retired football players. Fifteen retired players received daily supplementation of fish oil with cholecalciferol, antioxidants, natural vitamins and minerals, polysaccharides and phytosterol-amino acid complex for 6 months. Using an open-labeled repeated measures design, volunteers completed self-report assessment measures at baseline, 1, 3 and 6 months. Outcome measures were CDC HRQOL-4, WHOQOL-BREF, POMS, MFQ and pain self-assessment. General health rating improvement on CDC HRQOL-4 from month 1 was sustained to month 6 (p<0.0001). Mental health days improved at 6 months (p<0.05). WHOQOL-BREF showed increased health satisfaction at all measurement points (p<0.05) and the Physical and Psychological Domain Scores at 6 months (p<0.05). MFQ General Rating of Memory improved at 3 and 6 months (p<0.05). Vigor scale in POMS was significant at 3 months (p<0.05). Decreased pain was noted only for the elbow at month 1 and the knee at month 3 (p<0.05). No adverse events were reported. Results of this study offer preliminary insight into using dietary supplements to support and optimize quality of life in retired football players. Further research using a placebo-controlled design is needed to characterize the potential benefit to physical and psychological well-being of multiple dietary supplementations for this cohort.

## 1. Introduction

Football is a very popular sport that even a snowstorm will not deter avid fans from filling up a huge stadium (Mosier, 2011). The impact of the sport through injury and potential long term health effects is attracting increasing attention. For example, retired professional rugby league players may have at least one long term consequence of injuries sustained during their playing career (Meir, McDonald, & Russell, 1997). It is fairly known that when an individual plays football, he will sustain some kind of injury. The effects of this injury may even be magnified when participation factors such as BMI, playing position and time of injury are considered (Baron, Hein, Lehman, & Gersic, 2012). Many studies have reported that injuries may predispose athletes to osteoarthritis. A study of elite Australian football players showed a significant risk of both functional and radiological osteoarthritis related to a history of intra-articular or meniscal injury (Deacon, Bennell, Kiss, Crossley, & Brukner, 1997). The prevalence of osteoarthritis is significantly higher than for the general population (Drawer & Fuller, 2001) and that excess of early onset in males under age 60 among retired players may be due to the high incidence of injury in football (Golightly, Marshall, Callahan, & Guskiewicz, 2009). Similar early onset of osteoarthritis (OA) related to knee injuries has also been reported (Roos, 1998; Krajnc et al, 2010). Knee osteoarthritis was highly prevalent in male soccer players after an anterior cruciate ligament tear and resulting symptoms severely affected the knee related quality of life by middle age (von Porat, E. Roos, & H. Roos, 2004). The development of OA was associated with poorer outcomes on health related quality of life (HRQL) measures (Turner, Barlow, & Heathcote-Elliott, 2000).

There is widespread agreement that repeated blows to the head during a football player's career can result in brain damage (Bartholet, 2012). Professional football players are exposed to repetitive concussions and they may be at risk for cognitive impairment (Willeumier, Taylor, & Amen, 2012). The extent of concussion exposure was associated with increased memory complaints and over all post-concussion symptoms in a dose-dependent manner for retired and older recreational players (Thornton, Cox, Whitfiled, & Fouladi, 2008). In addition, recurrent sport-related concussion has also been related to an increased risk of clinical depression (Guskiewicz et al., 2005; Guskiewicz et al., 2007). Players self-reporting concussions throughout their career have been associated with a greater risk for depressive episodes later in life (Kerr, Marshall, Harding, & Guskiewicz, 2012).

Neurocognitive impairment due to concussion may also be extended to an increased risk of neurodegenerative diseases in retired athletes. A recent study showed that mortality rate due to neurodegenerative disease was three times higher in football players than the general US population (Lehman, Hein, Baron, & Gersic, 2012). Brain imaging research also showed that compared to a healthy control and active National Football League players, former National Football League players demonstrated decreased brain perfusion and EEG activation consistent with chronic brain trauma among professional football players (Amen et al., 2011b).

Chronic pain may also affect the quality of life of retired football players. Specifically, chronic pain and musculoskeletal disability, potentially acquired through injury and/or age-related decline, can interfere with physical activity and fitness during retirement, increase the risk of depression (Schwenk, Gorenflo, Dopp, & Hipple, 2007) and reduce quality of life. Consequently, as a group, retired professional football players are a population at risk of negative long term effects.

There is growing awareness by the public and the popular media (Raley, 2008; Associated Press, 2012) of the potential long-term health risks associated with retired football players, with affected former players publically discussing the controversial issue (Hendricks, 2012). Despite the public discussion and interest, there are a limited number of studies investigating the quality of life of retired football players and the potential for dietary interventions, through supplementation, to support beneficial health outcomes. Most published quality of life studies related to dietary supplements deal with diseased population, malnourished children, the elderly and active athletes. To date, there is only one published study related to dietary supplementation in 30 retired football players with traumatic brain injury. The study showed positive cognitive improvement following intake of multiple dietary supplements (Amen, Wu, Taylor, & Willeumier, 2011a). There is then a need to explore further how dietary interventions, specifically supplementation, may effect and support beneficial changes in this population group.

This exploratory open-label study aimed to determine whether dietary supplements can impact the quality of life of retired professional football players.

## 2. Materials and Methodology

### 2.1 Study Design

An open-label, repeated measures design was used to assess the effect of dietary supplementation with multiple nutrients over a six month period on quality of life outcome measures. Before initiation of the study, pre-screened, ambulatory and generally healthy volunteers were required to sign an Informed Consent form. After screening, participants were examined by a clinical staff to ensure that they are in general good health. Shoeless height and weight with minimal clothing were measured and the BMI was calculated. Blood pressure, pulse rate, past medical history and concomitant supplementations and medications were recorded. Blood samples were taken to assess complete blood count, liver and kidney function tests as a measure of overall health. Inclusion of participants in the 6 month intervention was based on the individual's clinical and laboratory blood measurements falling within the normal range for age. Throughout the study, participants were instructed not to alter their current diet or exercise regimen and not to take other dietary supplements other than the dietary supplements in the study. Before commencing the study, participants had a two week washout period after which they received the study products. Participants were given a tracking log sheet for each month to record their daily supplement intake, and any other health observations, including perceived adverse effects. Participants were required to return the log form at the end of each month as a measure of study compliance. Self-administered questionnaires to assess quality of life were completed on day 1 and at the end of 1, 3 and 6 months. Subjects were instructed to report any adverse signs or symptoms experienced while on the study products. The study protocol was reviewed and approved by an internal ethics committee to ensure the pilot study was conducted in compliance with the Good Clinical Practice (GCP) guidelines.

### 2.2 Subjects

A convenience sample of 21 generally healthy, ambulatory retired male, professional football players who were not involved in other research studies volunteered for study face-to-face pre-screening. Pre-screening exclusion criteria included: history of severe head/brain, neurological or psychiatric conditions, rheumatoid or other severe inflammatory joint conditions, gout, individuals with thyroid disorder, individuals with diabetes, individuals taking antidepressants, individuals taking Warfarin of other anti-coagulant medication, individuals who are lactose intolerant, participants who have just commenced a treatment regimen for arthritis, use of corticosteroids (intra-articular or systematic) within 4 weeks prior to baseline and throughout the study, liver function tests greater than 3 times the upper limit of normal at baseline confirmed by laboratory test by Sonora Quest Laboratories (Arizona), history of alcohol or substance abuse, history of allergy to iodine or shell fish, history of known contraindication or hypersensitivity to any of the ingredients of the supplements, participants who have participated in another clinical trial in the last 30 days, participants unwilling to comply with the study protocol and any other condition, which in the opinion of the investigators could compromise the study.

From the original sample of 21 volunteers who were deemed eligible and signed an Informed Consent form, 3 were eventually dropped before issuing the supplements because of non-compliance with protocol which included non-completion of baseline questionnaires, non-appearance for physical examination and no laboratory data on complete blood count, liver function and kidney function tests. Three more were excluded within a month because of low to no compliance with supplement intake and/or failure to complete and submit questionnaires at month 1, thus resulting to a final count of 15 subjects.

### 2.3 Supplements

The dietary supplements used in this study were selected to provide a low calorie, nutrient-rich profile of ingredients that have been shown to support optimal physiological function.

The participants were instructed to take daily the following supplements:1) 1 capsule 2 times a day of fish oil with cholecalciferol (Omega-3 with Vitamin D_3_ capsules) which contains 1110 mg of Omega-3 fatty acids (660 EPA and 450 DHA) with 830 IU of vitamin D_3._ 2) 1 tsp 2 times a day of 2 g/tsp of bulk polysaccharide powder (Ambrotose^®^ Complex powder) in food or juice consisting of a proprietary blend of Arabinogalactan (*Larix spp*.), aloe vera (inner leaf gel powder), ghatti gum, tragacanth gum, glucosamine HCL and rice starch. 3) 1 capsule 2 times a day of antioxidant (Ambrotose AO® capsule) that contained per capsule 18 IU vitamin E as mixed tocopherols; 113 mg of an antioxidant blend (quercetin dihydrate; grape skin extract; green tea extract; *Terminalia ferdinandiana* [Australian bush plum powder], 331 mg of a proprietary blend of plant polysaccharide and fruits and vegetables powders (aloe vera inner leaf gel, gum acacia, xanthan gum, gum tragacanth, ghatti gum, broccoli, Brussels sprouts, cabbage, carrot, cauliflower, garlic, kale, onion, tomato, turnip, papaya and pineapple. 4) 2 caplets 2 times a day of a phytosterol-amino acid complex (PLUS™ caplets) consisting of Wild Yam (root) 200 mg standardized to 12.5%, Diosgenin, 25 mg, Beta Sitosterol 25 mg, L-Arginine 95 mg, L-Glutamic Acid 200 mg, L-Lysine, 200 mg, Glycine 200 mg, Boron 1 mg, and 2.5 mg of the polysaccharide supplement Ambrotose. 5) 2 caplets 2 times a day of a natural vitamin and mineral supplement (PhytoMatrix^®^ caplets) consisting of Vitamin A as mixed carotenoids 2500 IU, Vitamin C 30 mg, Vitamin D 200 IU, Vitamin E 15 IU, Thiamin 0.75 mg, Riboflavin 0.8 mg, Niacin 8 mg, Vitamin B_6_ 1 mg, Folic Acid 260 mcg, Vitamin B_12_ 3 mcg, Biotin 75 mcg, Pantothenic Acid 3 mg, Calcium 255 mg, Iron 3mg, Iodine 75 mcg, Magnesium 5 mg, Zinc 7 mg, Selenium 80 mcg, Copper 0.8 mg, Manganese 1.2 mg, Chromium 120 mcg, Molybdenum 40 mcg, Sodium 10 mg, Boron 400 mcg, Vanadium 40 mcg, Aloe vera inner leaf gel powder 40 mg, Broccoli Concentrate 40 mg standardized to 6% Glucosinolate,2.4 mg sulforaphane 20 mcg, Cranberry Juice concentrate 40 mg standardized to 35% organic acids 14 mg, Grape skin extract 25 mg standardized to 50% polyphenols 20 mg, Rutin 40 mg.

The supplements used in the study were designed, developed and provided by Mannatech, Incorporated, Coppell, TX. The ingredients, source and quality, used in each supplement formulation are classified as GRAS (Generally Recognized as Safe) by the U.S. Food and Drug Administration and the finished products are NSF Certified according to the NSF/ANSI 173 Dietary Supplement Standard, the only American National Standard for dietary supplements. The NSF certification ensures that a product contains only the ingredients indicated on the label and is free of impurities, and that Good Manufacturing Practices (GMPs) were used in the manufacturing facility (NSF International, 2012).

### 2.4 Quality of Life Assessments

The following standard measurements were used: Healthy Days Core Module (CDC HRQOL-4), World Health Organization Quality of Life Questionnaire (WHOQOL-BREF), Profile of Mood States (POMS) and Memory Functioning Questionnaire (MFQ). The subjects completed the questionnaires on day 1 and at the end of months 1, 3 and 6.

a) The CDC HRQOL-4 (Centers for Disease Control, 2000) uses a set of questions called “Healthy Days Measure”. The first question asks about the general health if it is excellent, very good, good, fair or poor; the second and third questions about the number of days physical and mental health were not good in the past 30 days and the last question inquires about the number of days in the previous month did poor physical and mental health interfered with usual activities.
b) The WHOQOL-BREF (World Health Organization, 2004) produces a profile with four domain scores: physical health, psychological health, social relationships and environmental health and two overall QOL and two individually scored items about an individual's overall perception of quality of life and health. The four domain scores are scaled in a positive direction with higher scores indicating a higher quality of life.
c) The POMS (McNair, Lorr, & Droppleman, 1992) is a self-report questionnaire that contains 65 items pertaining to six mood states: tension-anxiety, depression-dejection, anger-hostility, vigor-activity, fatigue-inertia, and confusion-bewilderment. A composite of the 6-mood states was calculated to provide total mood disturbance score. Participants are asked to rate these on a 5-point scale (0= not at all to 4= extremely) indicating how they have felt during the past week including today.
d) The MFQ (Gilewski, Zelinski, & Schaie, 1990) evaluates perception of everyday memory functioning using four factors namely: General Frequency of Forgetting, Seriousness of Forgetting, Retrospective Functioning and Mnemonics Usage. The items under each factor are Likert scaled from 1 to 7, with the higher number representing a more positive response. The factor scores are calculated and higher scores indicating higher level of self- reported memory functioning, less problems related to forgetting or improved memory ability.

In addition to the standard questionnaires, the investigators developed a simple questionnaire adapted from a general health status survey by the sponsor (Mannatech, 1998) about the participants’ experience over the past 30 days of pain, limitation of range of movement, weakness and stiffness of extremities and selected body parts. Participants rated on a scale from 0 to 5 (from 0=does not apply to 5 = agree) how often they had experienced positive levels of these qualities.

All the questionnaires were self-administered after the investigators delivered and explained the contents of the questionnaires. O’Connor (1993) cites Bergner et al. (1981) who mentioned differences in the reliability of three types of administration of Sickness Impact Profile (SIP) questionnaires: a) interviewer-administered; b) interviewer delivered and explained, and then self-administered; and c) mail delivered and self-administered. The mail delivered, self-administered had the lowest internal consistency reliability (measured by Cronbach's Alpha), and also the lowest correlations with self-assessed dysfunction and illness (0.48 and 0.38 correspondingly). Interviewer delivered/self-administered produced the greatest correlations with self-assessment dysfunction and illness scores, these being 0.74 and 0.67 correspondingly. Interviewer administered questionnaires were in the middle, with correlations of 0.64 and 0.55.

The key outcome measures of CDC HRQOL-4, WHOQOL-BREF, POMS and MFQ are well validated tools that have been used in many studies. They are also easy to administer, show inter-rater reliability, and moderate to high correlation consistency between self and interviewer administered. They are considered generic and general QOL measures are proposed as better to be able to capture a variety of dysfunction not related to specific disease conditions (O’Connor, 1993).

### 2.5 Statistics

Analysis was conducted using SAS v.9.3 (SAS Institute, Cary, NC). Demographic and key characteristic variables were summarized using descriptive statistics. The percentages of subjects in each category were calculated for the categorical variables. Data logic checks, out of range values and internal inconsistencies were used to detect outliers and inconsistent data that may skew the results. All outcome measures were analyzed using a two-tailed paired t-test to compare specific time periods vs. baseline values. Results were considered to be statistically significant when the accompanying statistical test yields a two-tailed probability of less than 0.05.

## 3. Results

### 3.1 Subject Characteristics and Participation

Fifteen subjects completed the first and three months of the study, and 12 continued through the sixth month. The three participants who completed only up to three months voluntarily withdrew for personal reasons unrelated to the study; these include work commitments and unforeseen travel. The baseline characteristics of the participants are described in Table 1.

**Table 1:** Baseline characteristics of the study participants (N=15)

Variable	Mean (SD)	
Age (yrs)	49.60	(8.24)
Ht (cm)	188.52	(7.37)
Wt (kg)	125.55	(24.72)
BMI	35.17	(5.75)
Professional Football Career (yrs)	7.40	(3.20)

Dietary Supplement Use (%)		27.00
Self-Reported Concussions (%)		86.00
Self-Reported Injuries (%)		93.00
Ethnicity (%)		
White		33.33
African American		60.00
Other		6.67
Marital Status (%)		
Single		20.00
Married		73.33
Divorced/Separated		6.67
Employed (%)		
No		46.70
Yes		53.30

### 3.2 Effects of Supplementation on Quality of Life

There were significant findings in the key outcome measurements.

#### 3.2.1 The CDC HRQOL-4

The General Health has improved when asked to rate from poor to excellent starting at 1 month and sustained to 6 months (p<0.05). There were decreasing numbers of unhealthy days caused by poor physical and mental health. Mental health which includes stress, depression and problems with emotions had decreasing trend of “not good” days and statistically significant at 6 months (p<0.05) (Table 2). The number of unhealthy days that affected usual daily activities also decreased.

**Table 2:** CDC HRQOL-4 measures at 1, 3 and 6 months compared to baseline

Measure	Month	N	Mean Difference	SD	*P* value
General Health	1	15	-0.40	0.51	*0.0086
	3	15	-0.67	0.62	*0.0009
	6	12	-1.17	0.58	*<0.0001
Poor Physical Health	1	15	-0.33	14.40	0.9298
	3	15	-3.13	8.24	0.1628
	6	12	-4.33	9.61	0.1467
Poor Mental Health	1	15	-3.60	16.53	0.4133
	3	15	-4.27	13.38	0.2370
	6	12	-10.75	14.88	*0.0293
Poor Health Interfered with Usual Activities	1	15	-1.87	7.27	0.3368
	3	15	-3.07	6.33	0.0816
	6	12	-5.08	10.17	0.1112
Healthy Days	1	15	5.47	16.90	0.2309
	3	15	6.33	14.92	0.1224
	6	12	10.67	15.90	*0.0403

* p<0.05

**Figure 1:**
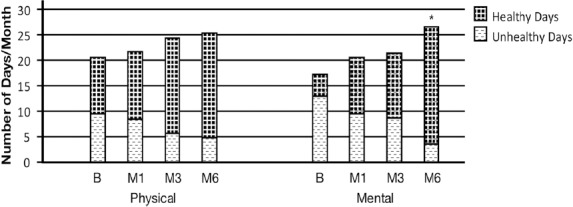
The number of improved physical and mental health days continually increased with mental health days significant at 6 months (*p<0.05)

In order to provide an accurate interpretation of the number of healthy and non-healthy days that impact daily activities, the following calculations were carried out. First, responses in questions 2 (physical health) and 3 (mental health) were combined to calculate a summary index of overall unhealthy days with a logical maximum of 30 unhealthy days. For example, as indicated by the CDC, a person who reports 4 physically unhealthy days and 2 mentally unhealthy days is assigned a value of 6 unhealthy days, and someone who reports 30 physically unhealthy days and 30 mentally unhealthy days is assigned the maximum of 30 unhealthy days. Second, a maximum overlap in the responses to questions 2 and 3 were combined. For example a person who reports 4 physically unhealthy days and 2 mentally unhealthy days is assigned a value of 4. Both calculations showed trends for a decrease in the number of unhealthy days reported by participants each month during the 6 months period. For calculation with maximum overlap, a statistically significant difference was observed at 6 months (p<0.05) as seen in Table 2. The number of healthy days, calculated by subtracting the number of unhealthy days from 30 days, increased (Figure 1).

#### 3.2.2 WHO-BREF QOL

Both the general rating of quality of life and satisfaction with health improved from baseline to month 6 (p<0.05). Three domains, Physical, Psychological and Environmental, showed positive direction and the mean QOL scores at all months compared to baseline were increasing and statistically significant at month 6 (p<0.05). There were no statistically significant findings on the Social domain (Table 3).

**Table 3:** WHOQOL-BREF measures at 1, 3 and 6 months compared to baseline

Variable	Month	N	Mean Difference	SD	*P* value
General Rating Quality of Life	1	15	-0.07	0.88	0.7744
	3	15	-0.07	0.59	0.6702
	6	12	0.33	0.49	*0.0388
Satisfaction with Health	1	15	0.60	0.91	*0.0230
	3	15	0.47	0.83	*0.0479
	6	12	1.00	0.74	*0.0007
Physical Health	1	15	0.80	10.29	0.7678
	3	15	2.40	15.23	0.5514
	6	12	11.83	15.76	*0.0247
Psychological Health	1	15	-0.80	14.03	0.8284
	3	15	2.20	13.47	0.5372
	6	12	10.00	14.91	*0.0403
Social Relationships	1	15	-2.40	13.37	0.4984
	3	15	2.27	15.17	0.5719
	6	12	-1.83	18.01	0.7310
Environmental Health	1	15	-3.87	9.79	0.1484
	3	15	1.47	8.65	0.5221
	6	12	6.33	9.87	*0.0480

* p<0.05

#### 3.2.3 MFQ

When asked to rate memory, the general rating of memory showed positive trend and statistically significant differences at months 3 and 6 (p<0.05) compared to baseline. On frequency of forgetting, there was also a positive trend but statistically significant only at month 3 (p<0.05). The other factors did not show statistically significant differences (data not shown).

#### 3.2.4 POMS

There was a significant difference on the Vigor scale, with a significant difference between time point 3 compared to baseline, showing that participants rated Vigor as significantly higher at 3 months compared to baseline (p<0.05). There were no other significant differences between time points compared to baseline, or between time points (data not shown).

#### 3.2.5 Pain, Range of Movement, Weakness and Stiffness of Extremities and Selected Body Parts

In relation to pain, there was decreasing trend in perceived pain of the knee at month 3 and a significant reduction in reported perceived pain of the elbow at month 1 (p<0.05). Although decreasing trends in problems with range of movement, weakness and stiffness in arm and legs were observed, these did not reach statistical significance (data not shown).

### 3.3 Adverse Effects

The supplements were well-tolerated by all of the participants in this study. There were no serious adverse effects reported, both perceived or that required a physician's care. Laboratory results of complete blood count, liver and kidney function tests were not clinically significant.

## 4. Discussion

This study aimed to determine preliminary effects of dietary supplementation on the quality of life of retired football players. The results of this study suggests that daily supplementation with fish oil and cholecalciferol, antioxidants, natural vitamins and minerals, polysaccharides and phytosterol-amino acid complex for 6 months can provide significant improvements in key QOL parameters, including increased number of healthy days, decreased perceived pain, and better vigor and memory functioning.

There is increasing evidence for the role of dietary supplementation in promoting better health outcomes and significant consumer belief in the benefits that supplementation might provide (Block et al., 2007; Egan, Hodgkins, Shepherd, Timotijevik, & Raats, 2011). However, there are few studies exploring the potential impact of dietary supplementation in retired football players. A published study done in 30 former NFL players with demonstrated brain damage and cognitive impairment investigated the effect of dietary supplementation with fish-oil, high-potency vitamin and other brain enhancement supplements for 6 months. The results showed improvements in cognitive and cerebral blood flow from baseline to measurement points (Amen et al., 2011a).

While the effect of specific nutrients was not explored in this study, the results indicate improvements in quality of life that are consistent with emerging evidence from previous research regarding the role nutrition on physical and psychological functions that underpin quality of life.

Oral polysaccharides have been shown to have the ability to promote brain function and behavior as well as immunomodulatory effects (Nelson, Ramberg, Best, & Sinnott, 2012; Ramberg, Nelson, & Sinnott, 2010). Specifically, oral intakes of plant-polysaccharides, as used in this study, have been shown to improve memory recall and psychological well-being across both objective and self-report measures in middle-aged adults. In a randomized, double-blind, placebo-controlled trial involving more than 100 healthy adults, individuals who consumed the same proprietary polysaccharide powder used in this study reported numerous health and well-being benefits following 12 weeks of intake, compared with subjects taking a placebo. Perceived benefits reported by individuals who consumed this included gastrointestinal effects (Best, Kemps, & Bryan, 2012). An earlier research involving 109 healthy middle-aged male and female adults showed that the polysaccharide powder (4 grams/day for 12 weeks) significantly improved memory and improved psychological well-being (Best, Kemps, & Bryan, 2010). The improved memory and better performance on other cognitive tasks associated with the use of the polysaccharide may be independent from blood glucose effects (Best, Howe, Bryan, Buckley, & Scholey, 2011). Another age group was also studied where a randomized, double-blind, placebo-controlled study investigated the impact of the polysaccharide on the brain function of 62 healthy young adults that resulted in significantly improved visual discrimination and working memory (Stancil & Hicks, 2009). Interestingly, consumption of the polysaccharide also enhanced brainwave frequencies known to be associated with attention or alertness (Wang, Szabo, & Dykman, 2004). The reported improvements in mental health (CDC HRQOL-4), psychological health (WHOQOL-BREF) and memory functioning (MFQ) found in this study may be related to intake of the polysaccharides as one of the many nutrients provided.

The benefit of antioxidant supplementation on health outcomes have become of increasing interest due to controversial findings of both beneficial and non-beneficial, even detrimental effects in humans of high intakes of antioxidant supplementation (Biesalski, Grune, Tinz, Zollner, & Blumberg, 2010; von Arnim et al., 2012). The antioxidant preparation used have been shown in a 21-day randomized, double-blind, placebo-controlled crossover trial of 25 healthy adults to significantly increase two measures of antioxidant capacity in the blood: oxygen radical absorbance capacity (ORAC) and Trolox Equivalent Antioxidant Capacity (TEAC) (Bloomer, Canale, Blankenship, & Fisher-Wellman, 2010). Two earlier open-label studies using the same preparation on healthy adults showed significant increases in serum ORAC, between 36.6% to 37.4%, compared with baseline (Boyd et al., 2003; Myers et al., 2010). Antioxidants were part of a regimen used in highly skilled athletes for effective improvement of athletic performance, recovery from fatigue after exercise and ward off immunodeficiency (Trushina, Mustafina, Nikitiuk, & Kuznetsov, 2012). It was proposed that antioxidants may have a role in the prevention of neurodegenerative disorders through both direct protection against oxidative stress and indirect protection thru suppression of glia-mediated inflammation (Wang, Wen, Huang, Chen, & Ku, 2006). It was also suggested that suppression on neuroinflammation processes associated with reactive oxygen species (ROS) and reactive nitric oxide species (RNS) maybe possible with antioxidants and nitrone-based free radical traps (Floyd, 1999). It is not clear how the antioxidants may have affected the health of the football players in the study but it could be due to interplay of some of the mechanisms presented.

The amount of omega-3 fatty acids used in the study is 1110 mg. The U.S. Food and Drug Administration (FDA) has determined that the use of EPA and DHA omega-3 fatty acids as dietary supplements is safe, provided that the daily intakes of EPA and DHA do not exceed 2 grams per day from dietary supplement sources such as fish oil (US Food and Drug Administration, 2004). Fatty acids are the building blocks of lipids, making them important sources of energy for the body and the main components of cell membranes. There is a number of known health benefits associated with omega-3 fatty acid intake. However, the human body cannot synthesize omega-3 fatty acids on its own so these nutrients must be provided by the diet (Institute of Medicine, 2005). The omega-3 fatty acids may have neuroprotective and regenerative potential in traumatic neurological injury (Michael-Titus, 2009). In a study of professional football players, omega-3 supplementation significantly improved the lipid profile of active players specifically increasing eicosapentaenoic acid and docosahexaenoic acid levels in plasma and was shown to be an effective approach to improve modifiable cardiovascular risk lipid factors in professional football players (Yates et al., 2009). Additional intervention studies with omega-3s demonstrated benefits of these nutrients in some inflammatory conditions using both animal and human models (Calder, 2012). Polyunsaturated fatty acids also play a vital role in pain regulation (Tokuyama & Nakamoto, 2011; Goldberg & Joel, 2007). The reported effects of pain reduction in the elbows and knees with the other perceived benefits reported by the retired football players in this study may be due to the effect of the omega-3 fatty acids.

The fat-soluble vitamin D3 (cholecalciferol) is synthesized by humans in the skin when exposed to ultraviolet-B (UVB) rays from sunlight. For Americans, the current average daily intakes of vitamin D are well below suggested adequate intakes (Mithal et al., 2009), and majority of the population in the world is deficient in this important vitamin (Palacios, 2006). Vitamin D regulates blood calcium and phosphorus concentrations by enhancing the absorption of these minerals in the small intestine. With the absorption of calcium, vitamin D therefore helps to form and maintain strong bones and teeth (Annweiler et al., 2010; Institute of Medicine, 2011). This nutrient also helps prevent falls and maintain physical performance in the elderly (van Etten & Mathieu, 2005; Annweiler et al., 2010). Immune and nervous system health can be maintained (Maggini, Wintergerst, Beveridge, & Hornig, 2007; McCann & Ames, 2008; Bertone-Johnson, 2009) and overall quality of life can be improved (Holick et al., 2011) with adequate vitamin D intake. In this study, the retired players had increased number of healthy days and at the same time reported that they were able to perform the usual daily activities better because of better physical and mental health.

The phytosterol-amino acid complex and the natural vitamins and minerals used in the study also demonstrated safety and beneficial effects in previous studies (Myers, Brooks, Rolfe, & O’Connor, 2008; Udani, 2008). Amino acids like arginine become useful under stressful conditions (Barbul, 1986). Lysine, an essential amino acid that must be supplied by the diet and mostly provided by protein intake, is probably the least abundant amino acid present in foods (The Merck Index, 1996). The use of amino acids and other supplements increased perceived energy and reduced subjective fatigue during strenuous exercise (Spradley et al., 2012). Dietary supplementation with one or a mixture of amino acids like arginine, cysteine, glutamine, leucine, proline, and tryptophan which are called functional amino acids because they regulate key metabolic pathways are necessary for maintenance, growth, reproduction, and immunity (Wu, 2009). Phytosterols are fats present in plants and because the human body cannot produce these, they must be obtained through the diet. Epidemiologic studies suggest that phytosterol intake supports good health (Awad & Fink, 2000). Wild yam extract, derived from *Dioscorea* species is reported to contain appreciable amounts of calcium and vitamin C (Coursey, 1965) as well as saponin and diosgenin (Mirkin, 1991). Extensive safety testing of *D. villosa* extracts did not cause adverse effects when consumed by adults in large amounts (*Int J Toxicol*, 2004). Many health benefits were also observed with the use of wild yam (Reavley, 1998). An interesting new finding using an animal model showed possible effects of diosgenin on the central nervous system where diosgenin promotes the differentiation of progenitor oligodentocyte cells into mature oligodendrocyte through activation pathway that accelerates remyelination (Xiao et al., 2012). The many uses and benefits of vitamins and minerals have been extensively discussed in literature (Combs, 1998; Institute of Medicine, 2000a; Institute of Medicine, 200b). The reported improvements in general, physical and mental health by the retired football players in this study may be related to consumption of the nutrients described.

The extensive number of potential mechanisms by which the nutrients contained in the supplements used might impact physical and psychological functions that underpin quality of life is beyond the scope of this manuscript. Although the preceding discussion presented the benefits of the dietary supplements and possible actions of the ingredients in the body, it is not the aim of this paper to discuss the specific mechanisms of actions by which the supplements may have affected the quality of life of the retired football players. However, a generic understanding of the role nutrition plays in supporting a number of physiological mechanisms has been discussed. The aim of the paper is to explore the role of multiple dietary supplements in affecting quality of life of a select group. It is noted that the baseline BMI (35.17) of the participants is high. However since the dietary supplements provided were not specifically designed for weight management, this factor was not identified as a key outcome measure of the study and therefore is not discussed here. It will be of great interest to conduct a placebo controlled or a comparative effectiveness study using similar and other supplements to see if the results of QOL improvement can be duplicated in a cohort with the same characteristics like BMI, age, time in professional sports and related variables.

## 5. Conclusion

This study provides novel, positive findings of general improvements in quality of life, satisfaction with health and positive impact on cognitive health among retired professional football players who used dietary supplements. Given the open-label design, no control group and small study population, cautious interpretation is required. Methodological considerations in the pilot characteristics of the study such as the free-living context for completion of the questionnaires compared to laboratory assessment over 6 months may have reduced the ability to detect whether behaviors and other life events external to the study affected the results. However, this is one of the few studies conducted on dietary supplementation and quality of life in retired football players using well-validated outcome measures.

While acknowledging the potential for self-report bias and social desirability bias in responses, the results of this study offer preliminary insight into using dietary supplement to support and optimize quality of life in retired players and lend support to research investigating the potential benefits of complex nutrition supplementation. Specifically, the results of this study indicate that daily intake of dietary supplements that included fish oil with cholecalciferol, antioxidants, natural vitamins and minerals, polysaccharides and phytosterol-amino acid complex can improve the quality of life of retired professional football players. In addition, supplementation was not associated with any adverse effects and the supplements were safe to consume over the 6 month time period.

Further research using randomized, double-blind, placebo-controlled design will extend knowledge of the potential benefits nutritional dietary supplementation may have in retired athletes and reduce the prevalence of poor health outcomes in this cohort.

### Conflicts of Interest

The study was sponsored and conducted, and the manuscript was prepared and submitted, by Mannatech, Incorporated that sells the supplements in the study. RAS and RLM are employees of Mannatech, Incorporated. Data and statistical analyses were performed independently by SB, faculty at the University of North Texas Health Science Center and TB, research fellow at the University of South Australia.
